# Marker-based linkage map of Andean common bean (*Phaseolus vulgaris* L.) and mapping of QTLs underlying popping ability traits

**DOI:** 10.1186/1471-2229-12-136

**Published:** 2012-08-09

**Authors:** Fernando J Yuste-Lisbona, Marta Santalla, Carmen Capel, Manuel García-Alcázar, María De La Fuente, Juan Capel, Antonio M De Ron, Rafael Lozano

**Affiliations:** 1 Departamento de Biología Aplicada (Genética), Edificio CITE II-B, Centro de Investigación en Biotecnología Agroalimentaria (BITAL). Campus de Excelencia Internacional Agroalimentario, CeiA3, Universidad de Almería, Carretera de Sacramento s/n, 04120, Almería, Spain; 2 Departamento de Recursos Fitogenéticos, Misión Biológica de Galicia-CSIC, P.O. Box 28, 36080, Pontevedra, Spain

## Abstract

**Background:**

Nuña bean is a type of ancient common bean (*Phaseolus vulgaris* L.) native to the Andean region of South America, whose seeds possess the unusual property of popping. The nutritional features of popped seeds make them a healthy low fat and high protein snack. However, flowering of nuña bean only takes place under short-day photoperiod conditions, which means a difficulty to extend production to areas where such conditions do not prevail. Therefore, breeding programs of adaptation traits will facilitate the diversification of the bean crops and the development of new varieties with enhanced healthy properties. Although the popping trait has been profusely studied in maize (popcorn), little is known about the biology and genetic basis of the popping ability in common bean. To obtain insights into the genetics of popping ability related traits of nuña bean, a comprehensive quantitative trait loci (QTL) analysis was performed to detect single-locus and epistatic QTLs responsible for the phenotypic variance observed in these traits.

**Results:**

A mapping population of 185 recombinant inbred lines (RILs) derived from a cross between two Andean common bean genotypes was evaluated for three popping related traits, popping dimension index (PDI), expansion coefficient (EC), and percentage of unpopped seeds (PUS), in five different environmental conditions. The genetic map constructed included 193 loci across 12 linkage groups (LGs), covering a genetic distance of 822.1 cM, with an average of 4.3 cM per marker. Individual and multi-environment QTL analyses detected a total of nineteen single-locus QTLs, highlighting among them the co-localized QTLs for the three popping ability traits placed on LGs 3, 5, 6, and 7, which together explained 24.9, 14.5, and 25.3% of the phenotypic variance for PDI, EC, and PUS, respectively. Interestingly, epistatic interactions among QTLs have been detected, which could have a key role in the genetic control of popping.

**Conclusions:**

The QTLs here reported constitute useful tools for marker assisted selection breeding programs aimed at improving nuña bean cultivars, as well as for extending our knowledge of the genetic determinants and genotype x environment interaction involved in the popping ability traits of this bean crop.

## Background

Popbean or nuña bean (*Phaseolus vulgaris* L., Fabaceae) is traditionally grown in the Andean highlands of South America at 2,000-3,000 meters above sea level, where it is commonly sold in local markets or consumed at home, and it is thought to be an ancient pre-ceramic landrace [1]. It seems probable that nuña beans originated in the Andes, where they are sympatric with wild and primitive common bean populations in certain parts of Peru and Bolivia, and they may have been present in the early stages of Andean agriculture [2,3]. The first selection pressures leading to domestication of common bean could have resulted in the development of popping beans, and it appears that toasting grains was a well-established tradition in the Andes and possibly in Mesoamerica, where early maize races have also been used for popping. However, no evidence of nuña beans has been found in Mesoamerica, most likely due to genetic differences between the Mesoamerican and Andean gene pools [1]. This, among other factors, may explain their contrasting popping ability and photoperiod response [4].

Popbean is tropical in appearance, with aggressive indeterminate type 4 growth habit [5] and day-length sensitivity since it requires nights of at least 11–12 h for flowering induction. Growing nuña bean in temperate areas requires the development of cultivars that are insensitive to photoperiod, like the modern dry bean cultivars. Furthermore, with a view to commercial production, determinate growth habit is desirable, since it reduces growing and harvesting costs. The foremost trait that distinguishes popbean from all other types of bean is the ability to expand the cotyledons after grains explode in response to heating, which is referred to as popping expansion, similar to popcorn, although the popping mechanism is different. In popcorn, the endosperm is liquefied and explosive pressure builds up in the pericarp [6,7], and both pericarp thickness and endosperm starch type have been attributed to the popping ability. Thus, there are two crucial factors that influence the popping of popcorn: whether a kernel can pop or not, and if so, to what extent. Popping rate and flake size seemingly correspond to these two factors, which together determine high popping volume [8]. In contrast, popping in nuña beans is the result of pressurized steam trapped within and between the mesophyll cells in the cotyledons [9,10]. The nutritive value analysis of nuña bean revealed that they have a lower mean content of protein, phosphorus, iron, and boron than dry bean varieties, and a higher level of copper and starch, which may be related to their unique texture and taste similar to roasted peanuts. Lectins and other anti-nutritional compounds were higher in raw and boiled nuña samples than in toasted nuñas, while tannin levels did not change from raw to toasted treatments. Overall in-vitro digestibility was slightly lower for toasted nuñas than boiled dry bean [11]. Taken together, the nutritional features of toasted nuña beans make them a healthy snack, although commercial production would require the genetic improvement of other agronomic traits, particularly the day-length sensitivity that has likely restricted production and commercialization of nuña beans in temperate regions [12,13].

In maize, experimental evidence indicate that popping characteristics are quantitatively inherited [14,15], controlled by multiple genes [15-17], and influenced by environmental effects [18] and popping methods [19]. Both additive and dominant genetic effects play very important roles in the inheritance of popping characteristics [14], and several putative quantitative trait loci (QTLs) have been identified [8,20,21]. However, most of these studies on popping ability have focused on the maize crop, and there is little information on the ability to contribute towards popping expansion of the nuña bean. The identification of genomic regions associated with this popping ability would enable breeders to develop improved cultivars using marker assisted selection (MAS). To identify these genomic regions, it is important not only to establish accurate phenotyping methods, but also to develop a saturated molecular marker-based genetic linkage map, and then to detect QTLs associated with these popping traits. Molecular markers have emerged as powerful tools not only for mapping genes/QTLs governing economically important traits in crops [22], but also for unlocking the useful genetic diversity from unadapted/wild/unrelated germplasm [23].

Common bean is a diploid species with a genome size estimated at 450 to 650 Mb [24] that is distributed among 22 chromosomes (n = 11). The first core genetic linkage map of common bean was based on a recombinant inbred line (RIL) population resulting from the cross between representatives of the Mesoamerican (BAT93) and the Andean (Jalo EEP558) gene pools, which included 194 restriction fragment length polymorphic (RFLP) markers [25]. Nowadays the availability of microsatellite markers and their potential for anchoring new genetic maps have allowed a new expanded version of the core linkage map to be created with several hundred of these types of markers, including markers with putative gene functions. Thus, the current core common bean linkage map covers a genetic distance of 1258.8 cM and includes a total of 413 loci placed across 11 linkage groups (LGs) with an average distance between neighboring loci of 3.0 cM [26]. In addition, about fifteen RIL mapping populations and more than twenty five linkage maps have been developed, most of them created from inter-gene pool crosses, which include divergent parents showing high genetic polymorphism [27,28]. Nevertheless, few populations have been generated from intra-gene pool crosses [29-35], likely due to their low polymorphism level [36,37] although this kind of population does not usually show phenotypic abnormalities or undesirable segregating individuals [38,39]. Recently, a Mesoamerican saturated intra-gene pool map has been constructed by combining the genetic information from intra- and inter-gene pool segregating populations [35].

Mapping QTLs is of great importance to understand the genetic architecture underlying complex traits. Popping expansion seems to involve several genes, environmental factors, and gene-gene and gene-environment interactions. Therefore, to better understand the genetic control of popping expansion, in addition to detecting single-locus QTL effects, there is a need to identify the interactions among the different loci (epistatic QTLs, E-QTLs), as well as their environment interaction effects (QTLs x Environment, QE; and E-QTLs x Environment, E-QE). Indeed, these interactions have been successfully analysed for other complex traits in several crops species e.g. rice [40], wheat [41], cotton [42] or maize [43].

In order to identify genetic determinants of popping ability traits in common bean, we have evaluated the popping dimension index (PDI), expansion coefficient (EC) and percentage of unpopped seeds (PUS). The first two traits reflect structural changes of seeds when heating (cotyledon expansion), while the third is of considerable importance for breeding and from a commercial point of view. Besides, we have constructed a genetic map from an Andean RIL population, which includes 193 molecular markers. This has permitted the location of single-locus and epistatic QTLs involved in PDI, EC and PUS, some of which co-localized in four LGs. Markers associated to these QTLs could be used as genetic tools for MAS programs devoted to popping improvement of nuña bean cultivars.

## Results

### Phenotypic variation of popping traits and correlations in RIL population

Mean values, standard deviations and ranges of variation for popping traits are shown for each environment in Table 1. PDI, EC and PUS were clearly different between both parents and varied significantly among RILs. In addition, these popping traits showed a pattern of continuous distribution, which weakly departed from normality (data not shown). Strong transgressive segregations were observed for these traits, indicating that alleles with positive effects are distributed among the parents. The broad sense heritability value of EC was rather low (0.33 ± 0.04), whereas the heritability values of PDI (0.53 ± 0.04) and PUS (0.48 ± 0.04) were moderate, suggesting that genetic gains could be obtained when selecting for these traits. The phenotypic correlations between PUS and two popping traits showed that PUS was significant and negatively correlated with PDI and EC, while EC was significant and positively correlated with PDI in all environments assayed (Table 2). Highly significant differences were found among genotypes and genotype x environment interactions for all popping traits (Table 3).

**Table 1:** Mean values of the popping traits analysed in the RIL population PMB0225 x PHA1037

**Trait**	**Environment**		**Parents**			**RILs**	
		**PMB0225**	**PHA1037**	**P**_**par**_^**a**^_**.**_	**Mean**^**b**^	**Range**	**P**_**RIL**_^**a**^
Popping dimension index (PDI)	LD09	−0.99 ± 3.28			2.45 ± 7.69	−16.21 – 45.08	**
	SD09	−0.05 ± 1.44	22.29 ± 6.18	**	3.53 ± 6.17	−20.83 – 26.37	**
	LD10	−0.60 ± 0.30			2.77 ± 7.30	−21.95 – 39.91	**
	SD10	−1.17 ± 5.37	27.84 ± 4.81	**	1.21 ± 5.53	−10.44 – 28.96	**
	LD11	1.01 ± 1.35			3.28 ± 7.75	−26.57 – 31.88	**
Expansion coefficient (EC)	LD09	2.25 ± 2.63			11.82 ± 19.22	0.00 - 95.00	**
	SD09	7.15 ± 10.12	68.70 ± 22.23	**	24.89 ± 37.87	−53.33 - 383.33	**
	LD10	10.19 ± 9.17			13.37 ± 21.38	0.00 – 166.67	**
	SD10	3.33 ± 4.35	45.83 ± 9.48	**	8.96 ± 16.08	−28.21 - 105.00	**
	LD11	17.78 ± 1.92			19.65 ± 24.87	0.00 - 150.00	**
Percentage of unpopped seeds (PUS)	LD09	95.04 ± 4.18			84.41 ± 25.58	0.00 – 100.00	**
	SD09	95.00 ± 7.07	24.44 ± 19.44	**	52.85 ± 31.78	0.00 – 100.00	**
	LD10	77.78 ± 6.29			73.12 ± 28.19	0.00 – 100.00	*
	SD10	100.00 ± 0.00	14.00 ± 0.00	**	86.93 ± 27.18	0.00 – 100.00	**
	LD11	97.00 ± 5.24			77.42 ± 29.99	0.00 – 100.00	**

^a^ *, ** Significant differences between the two parents (par.) or among lines (RILs) at *P ≤* 0.05 and *P ≤*0.001, respectively.

^b^ Mean of 2 and 3 replicates ± standard deviation.

No data taken for popping traits in the parent PHA1037 under long-day conditions.

**Table 2:** Phenotypic correlations among the popping traits evaluated in the RIL population PMB0225 x PHA1037

**Trait**	**Environment**	**PDI**	**EC**
**EC**	LD09	0.76**	
	SD09	0.53**	
	LD10	0.75**	
	SD10	0.76**	
	LD11	0.79**	
**PUS**	LD09	−0.80**	−0.75**
	SD09	−0.68**	−0.44**
	LD10	−0.64**	−0.53**
	SD10	−0.86**	−0.78**
	LD11	−0.85**	−0.84**

*, ** Significant correlation at P ≤0.05 and P ≤0.001, respectively.

PDI, popping dimension index; EC, expansion coefficient; PUS, percentage of unpopped seeds.

**Table 3:** ANOVA for the popping traits measured in the RIL population PMB0225 x PHA1037

**Source of variation**	**Degree of freedom**	**PDI**	**EC**	**PUS**
Environment	4	367.95**	8648.06*	28427.43**
Block (Environment)	4	44.31*	901.49	1703.72**
Genotype	186	188.53**	1291.77**	2406.78**
Genotype x Environment	602	41.80**	593.09**	562.39**
Error	865	21.33	387.05	392.90

*, ** Significant differences at *P ≤* 0.05 and *P ≤* 0.001, respectively.

PDI, popping dimension index; EC, expansion coefficient; PUS, percentage of unpopped seeds.

### Genetic map construction

**Figure 1:**
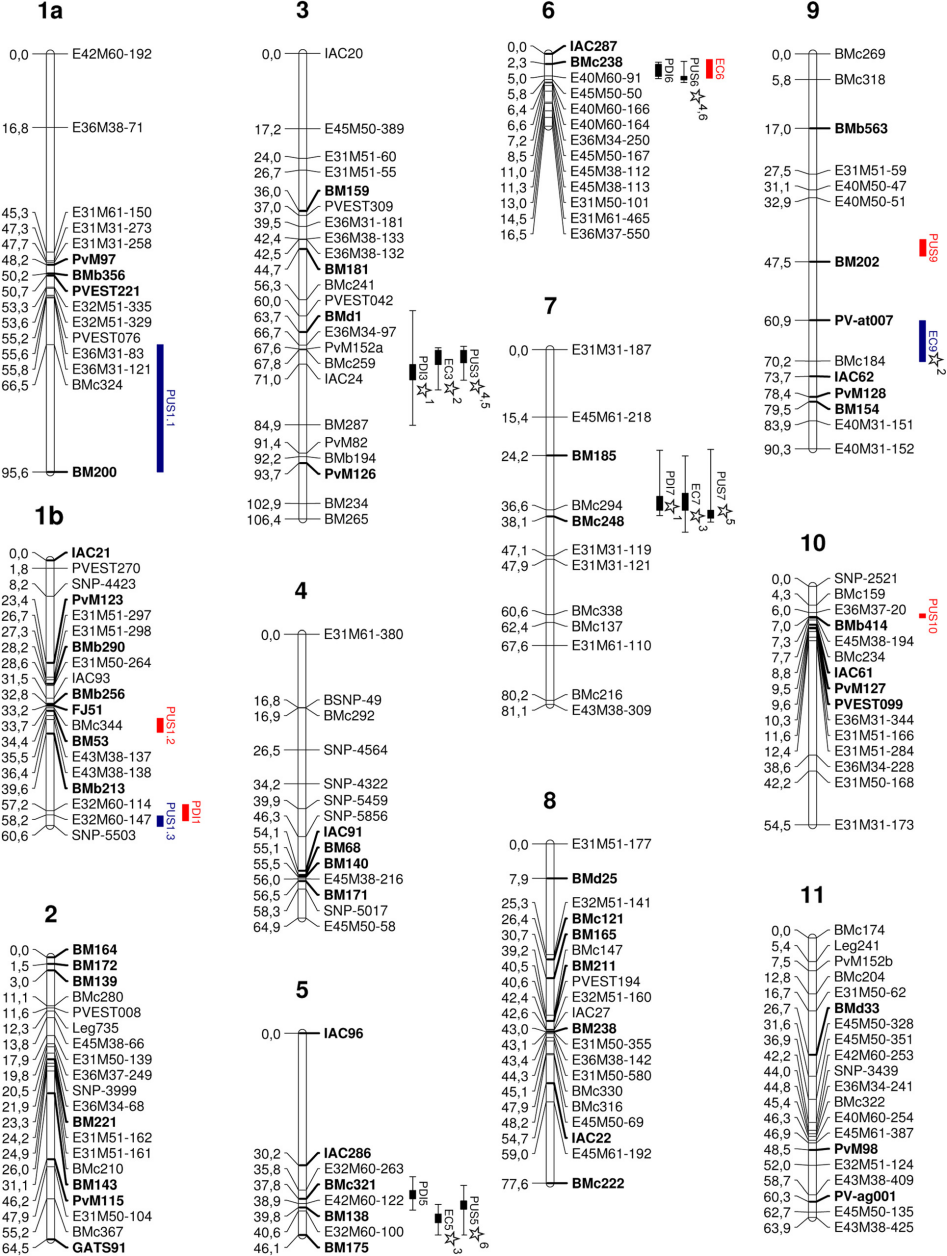
**Genetic linkage map of common bean based on the RIL population PMB0225 x PHA1037.** Location of single-locus QTLs and E-QTLs controlling popping traits: popping dimension index (PDI), expansion coefficient (EC), and percentage of unpopped seeds (PUS). Common SSR markers to previously published maps (see text for references) are indicated in bold. Names of QTLs are listed in Tables 5, 6, and 7. Cumulative distances among markers are indicated in cM to the left of the linkage group, names of markers are shown on the right. QTLs are depicted as vertical bars to the right of the linkage groups. QTLs detected by both MapQTL and QTLNetwork software packages are indicated in black, QTLs identified only by MapQTL are shown in red, and QTLs detected only by QTLNetwork are represented in blue. Epistatic interactions between QTLs are represented with numbered stars.

We screened PMB0225 and PHA1037 parents for DNA polymorphism by using amplified fragment length polymorphic (AFLP), simple sequence repeat (SSR) and single nucleotide polymorphism (SNP) markers. A total of 92 AFLP primer combinations were assayed, which allowed the amplification over 3,700 AFLP fragments, 279 (7.5%) of which were polymorphic between the parents. According to their inheritance pattern and reliability features, 18 combinations, producing 94 polymorphic loci, were selected for genotyping the RIL population. Furthermore, 1035 SSR and 251 SNP markers were screened, which rendered polymorphism rates of 10.2% and 7.2%, respectively. Thus, 106 SSR and 18 SNP polymorphic loci were also analysed in the RIL population for map construction. Some of the polymorphic markers (9 AFLPs, 11 SSRs and 5 SNPs) were not linked with any other marker in the existing map and so they could not be mapped. Finally, the genetic map developed from the cross PMB0225 x PHA1037 (Figure 1) was constructed with a total of 193 loci (85 AFLP, 95 SSR, and 13 SNP markers), of which 101 were dominant and 92 codominant, resulting in the formation of 12 LGs. These LGs were designated according to Pedrosa-Harand et al. [44] on the basis of 55 previously mapped common SSR markers [26,31-33,45-50], which were used as a guide for the assignment of LG number and orientation. The map spanned a total genetic distance of 822.1 cM, with an average of 68.5 cM per LG, ranging from 16.5 cM (LG 6) to 106.4 cM (LG 3). The density of markers ranged from 1.3 cM (LG 6) to 6.75 cM (LG 7), with an average of 4.3 cM per LG. A detailed description of this map is provided in Figure 1 and Table 4.

**Table 4:** Distribution of molecular markers on the linkage map constructed from the RIL population PMB0225 x PHA1037

**Linkage groups**	**Map length (cM)**	**No. of markers**	**Marker density (cM/marker)**		**Marker types**	
				**AFLP**	**SSR**	**SNP**
1a	95.6	15	6.4	9	6	-
1b	60.7	19	3.2	7	10	2
2	64.5	20	3.2	7	11	2
3	106.4	23	4.6	7	16	-
4	64.9	14	4.6	3	5	6
5	46.1	8	5.8	3	5	-
6	16.5	13	1.3	11	2	-
7	81.1	12	6.8	6	6	-
8	77.6	20	3.9	8	12	-
9	90.3	14	6.5	5	9	-
10	54.5	15	3.6	8	6	1
11	63.9	20	3.2	11	7	2
Total	822.1	193	4.3	85	95	13

### Single environment QTL analysis of popping traits

QTL analysis based on MQM mapping using MapQTL was undertaken to identify single-locus QTLs in individual environments; thus, a total of sixteen QTLs for popping traits were detected (Figure 1). Five single-locus QTLs were identified for PDI, of which one (PDI3^PP^) was identified in four environments, two were detected in two environments (PDI5^PP^ and PDI7^PP^), and the other two were detected in only one environment (PDI1^PP^ and PDI6^PP^). The percentage of phenotypic variation explained by a single QTL identified for PDI ranged from 7.5 (for PDI3^PP^ in LD09) to 18.7% (for PDI6^PP^ in SD10). For EC, two QTLs were detected in two or more environments (EC5^PP^ and EC7^PP^) and two QTLs were identified in only one environment (EC3^PP^ and EC6^PP^); in SD09 no significant QTLs were detected. The percentage of phenotypic variance for EC ranged from 7.3 (for EC7^PP^ in LD09) to 15.9% (for EC6^PP^ in SD10). In the case of PUS, seven single-locus QTLs were identified, four of which were detected in two environment conditions (PUS3^PP^, PUS5^PP^, PUS6^PP^, and PUS7^PP^) and three in only one environment (PUS1.2^PP^, PUS9^PP^, and PUS10^PP^). The percentage of phenotypic variance for PUS ranged from 6.3 (for PUS1.2^PP^ and PUS10^PP^, both detected in LD11) to 16.5% (for PUS6^PP^ in SD10). For each popping trait, QTLs with positive (alleles from PHA1037) and negative (alleles from PMB0225) additive values were identified. A complete description of the MQM mapping analysis is provided in Table 5.

**Table 5:** Single-locus QTLs detected for popping traits using multiple-QTL model mapping for individual environment analysis

**Trait Environment**	**QTL (position)**^**a**^	**Linkage group**	**Closest marker (position)**^**b**^	**LOD score**^**c**^	**LOD threshold**^**d**^	**R**^**2**^^**e**^	**A**^**f**^
Popping dimension index (PDI)							
LD09	**PDI3**^**PP**^ (68.1-74.5)	3	IAC24 (71.0)	2.7	2.6	7.5	1.92
	**PDI5**^**PP**^ (32.8-38.4)	5	BMc321 (37.8)	4.3		13.4	2.61
SD09	PDI1^PP^ (55.6-59.3)	1b	E32M60-147 (58.2)	3.0	2.8	7.7	1.57
	**PDI3**^**PP**^ (62.8-66.3)	3	BMd1 (63.7)	4.4		12.6	1.97
LD10	**PDI3**^**PP**^ (58.7-65.8)	3	PVEST042 (60.0)	3.1	2.6	8.2	1.93
	**PDI7**^**PP**^ (33.4-37.8)	7	BMc294 (36.6)	4.7		12.9	−2.44
SD10	**PDI3**^**PP**^ (62.1-65.9)	3	BMd1 (63.7)	4.7	2.7	9.2	1.46
	PDI6^PP^ (1.8-5.5)	6	E40M60-91 (5.0)	8.9		18.7	2.15
LD11	**PDI5**^**PP**^ (35.9-40.4)	5	BM138 (39.8)	3.0	2.8	7.7	2.07
	**PDI7**^**PP**^ (22.9-37.6)	7	BMc294 (36.6)	5.1		13.3	−2.72
Expansion coefficient (EC)							
LD09	**EC5**^**PP**^ (41.3-46.1)	5	BM175 (46.1)	3.6	2.6	9.8	6.18
	**EC7**^**PP**^ (30.1-37.7)	7	BMc294 (36.6)	2.7		7.3	−5.25
SD09	*Not significant*				2.1		
LD10	**EC7**^**PP**^ (30.5-37.9)	7	BMc294 (36.6)	3.5	2.4	10.7	−6.62
SD10	EC3^PP^ (67.2-76.8)	3	IAC24 (71.0)	5.9	2.6	12.0	5.69
	EC6^PP^ (1.2-5.4)	6	E40M60-91 (5.0)	7.6		15.9	6.75
LD11	**EC5**^**PP**^ (39.1-43.3)	5	BM138 (39.8)	4.2	2.8	9.8	6.82
	**EC7**^**PP**^ (32.7-41.6)	7	BMc294 (36.6)	5.9		14.2	−8.18
Percentage of unpopped seeds (PUS)							
LD09	**PUS5**^**PP**^ (38.3-46.1)	5	BM175 (46.1)	3.9	2.9	12.3	−9.14
SD09	**PUS3**^**PP**^ (66.9-74.6)	3	PvM152a (67.6)	3.3	2.9	8.9	−9.11
	PUS9^PP^ (42.3-46.1)	9	E31M51-59 (27.5)	3.4		10.1	9.78
LD10	**PUS6**^**PP**^ (3.1-6.4)	6	E40M60-91 (5.0)	3.2	2.9	9.7	−8.63
	**PUS7**^**PP**^ (22.7-38.4)	7	BM185 (24.2)	3.0		8.9	7.81
SD10	**PUS3**^**PP**^ (67.1-70.6)	3	PvM152a (67.6)	4.5	2.8	8.8	−8.26
	**PUS6**^**PP**^ (1.6-6.2)	6	BMc238 (2.3)	7.7		16.5	−11.36
LD11	PUS1.2 ^PP^ (35.9-39.1)	1b	E43M38-138 (36.4)	3.3	2.8	6.3	−7.23
	**PUS5**^**PP**^ (38.1-40.2)	5	BM138 (39.8)	4.6		8.7	−8.33
	**PUS7**^**PP**^ (26.9-39.3)	7	BMc294 (36.6)	5.2		9.9	9.29
	PUS10^PP^ (6.2-7.1)	10	BMb414 (7.0)	3.4		6.3	−6.79

Those QTLs identified in 2 or more environments are marked in bold.

^a^ QTL name (according to Miklas and Porch 2010) and its estimated map position (in Kosambi cM).

^b^ Nearest marker to peak of the detected QTL and its map position (in Kosambi cM).

^c^ LOD score detected for the nearest marker.

^d^ LOD thresholds score determined by permutation test for each trait in each environment (P = 0.05).

^e^ Percentage of the total phenotypic variation explained by the QTL.

^f^ Estimated additive effect. Positive values indicate allele arising from PHA1037 and negative values indicate allele arising from PMB0225.

### Multiple environment QTL analysis of popping traits

In addition to the single-locus QTLs identified for each environment, a single-locus QTL analysis for multi-environment was also carried out by using QTLNetwork. As the result of one-dimensional MCIM analysis, a total of fourteen single-locus QTLs were detected with significant genetic main effects and/or QTLs x Environment (QE) effects (Figure 1). Four single-locus QTLs were detected for PDI, three of which showed only individual additive effects (PDI3^PP^, PDI5^PP^, and PDI6^PP^); and the remaining QTL (PDI7^PP^) had both individual additive and QE interaction effects. The percentage of phenotypic variation explained for PDI by a single QTL ranged from 2.2 (for PDI6^PP^) to 7% (for PDI3^PP^); in the case of QTL PDI7^PP^, the phenotypic variation explained by QE interaction effects ranged from 0.6 (in SD09) to 1.6% (in SD10). For EC, four single locus QTLs were detected, two of which displayed only individual additive effects (EC3^PP^ and EC5^PP^) while the other two showed both individual additive and QE interaction effects (EC7^PP^ and EC9^PP^). The percentage of phenotypic variance for EC ranged from 2.8 (for EC7^PP^) to 4.6% (for EC5^PP^); the phenotypic variation explained by QE interaction effects ranged from 0.8 (for EC9^PP^ in SD10) to 1.4% (for EC7^PP^ in SD10). Six QTLs were identified for PUS, two of which had only individual additive effects (PUS3^PP^ and PUS5^PP^) while the other four displayed both individual additive and QE interaction effects (PUS1.1^PP^, PUS1.3^PP^, PUS6^PP^, and EC7^PP^). The percentage of phenotypic variance for PUS ranged from 0.6 (for PUS1.3^PP^) to 7.4% (for PUS3^PP^); the phenotypic variation explained by QE interaction effects ranged from 0.8 (for PUS1.1^PP^ in SD10) to 1.8% (for PUS1.3^PP^ in SD10). For the three popping traits, single-locus QTLs with positive and negative additive values were detected, indicating that alleles from both parents, PHA1037 and PMB0225, have a positive agronomical effect on the traits. A complete report of the one-dimensional genome scan analysis using QTLNetwork is given in Table 6.

**Table 6:** Single-locus QTLs and QTLs x Environment (QE) effects detected for the popping traits using multi-environment analysis

**QTL**	**Marker interval**	**LG (position)**^**a**^	**A**^**b**^	***h***^**2**^**(a)**^**c**^	**QE AE**^**d**^	***h***^**2**^**(ae)**^**e**^
Popping dimension index (PDI)						
PDI3^PP^	IAC24-BM287	3 (71.0-84.9)	3.74^***^	7.0	ns	
PDI5^PP^	E32M60-263-BMc321	5 (35.8-37.8)	1.31^***^	6.9	ns	
PDI6^PP^	BMc238-E40M60-91	6 (2.3-5.0)	1.46^***^	2.2	ns	
PDI7^PP^	BM185-BMc294	7 (24.2-36.6)	−1.75^***^	6.1	1.01^*^ AE2 1.41^**^ AE4 −1.44^**^ AE5	0.6 1.6 1.4
Expansion coefficient (EC)						
EC3^PP^	BMc259-IAC24	3 (67.8-71.0)	3.67^***^	4.3	ns	
EC5^PP^	E32M60-100-BM175	5 (40.6-46.1)	4.08^***^	4.6	ns	
EC7^PP^	BM185-BMc294	7 (24.2-36.6)	−6.14^***^	2.8	5.77^**^ AE4 −4.58^*^ AE5	1.4 0.9
EC9^PP^	PV-at007-BMc184	9 (60.9-70.2)	3.39^***^	3.4	−2.75^*^ AE4	0.8
Percentage of unpopped seeds (PUS)						
PUS1.1^PP^	BMc324-BM200	1a (66.5-95.6)	10.29^***^	1.6	−10.92^*^ AE4	0.8
PUS1.3^PP^	E32M60-147-SNP-5503	1b (58.2-60.6)	−7.01^***^	0.6	−8.29^**^ AE2 7.39^**^ AE4	1.6 1.8
PUS3^PP^	BMc259-IAC24	3 (67.8-71.0)	−11.51^***^	7.4	ns	
PUS5^PP^	BM138-E32M60-100	5 (39.8-40.6)	−8.04^***^	6.5	ns	
PUS6^PP^	E40M60-91-E45M50-50	6 (5.0-5.8)	−8.69^***^	4.8	−3.85^**^ AE4 3.58^*^ AE5	1.6 1.1
PUS7^PP^	BMc294-BMc248	7 (36.6-38.1)	4.99^***^	3.1	−3.17^*^ AE4 3.51^*^ AE5	1.2 1.1

^a^ Linkage group and the estimated confidence interval of QTL position in brackets (in Kosambi cM).

^b^ Estimated additive effect. Positive values indicate that alleles from PHA1037 have a positive effect on the traits, and negative values indicate that positive effect on the traits is due to the presence of the alleles from PMB0225.

^c^ Percentage of the phenotypic variation explained by additive effects.

^d^ Predicted additive by environment interaction effect. AE1, AE2, AE3, AE4, and AE5 additive by environment interaction effect associated with environments LD09, SD09, LD10, SD10, and LD11, respectively. The meaning of sign values is described in the second footnote (^b^).

^f^ Percentage of the phenotypic variation explained by additive x environment interaction effect.

^*^P ≤ 0.05, ^**^P ≤ 0.01, ^***^P ≤ 0.001. Only significant effects are listed. ns = No significant effects on the five environmental conditions evaluated.

### Epistatic and environmental interactions

Given that popping expansion seem to be a complex polygenic trait, genetic interactions may have significant effects on the phenotypic values. Therefore, two-dimensional genome scan was undertaken for multi-environment analysis using QTLNetwork to identify epistatic and environment interactions among QTLs. A total of ten epistatic QTLs (E-QTLs) involved in six epistatic interactions (Table 7 and Figure 1) were detected for the three evaluated traits. The percentage of phenotypic variance explained by the interaction of these E-QTLs was low, ranging from 0.2 (for EC) to 2.7% (for PDI). Interestingly, the ten E-QTLs identified were previously detected as single-locus QTLs, which indicated that these QTLs not only participated in epistatic interactions, but they also had an individual effect. Regarding PDI, only one epistatic interaction was detected, between QTLs E-PDI3^PP^ and E-PDI7^PP^, explaining 2.7% of phenotypic variation. For EC, two epistatic interactions were identified involving four E-QTLs; the percentage of phenotypic variance explained by the interactions of E-EC3^PP^-E-EC9^PP^ and E-EC5^PP^-E-EC7^PP^ was 0.2% and 2.6%, respectively. E-QTL x Environment (E-QE) interaction effects were not significant for either PDI or EC popping traits. In the case of PUS, three epistatic interactions and four E-QTLs were detected, and two of the interactions showed only genetic effects and explained 0.6% (E-PUS3^PP^-E-PUS7^PP^) and 1.8% (E-PUS5^PP^-E-PUS6^PP^) of phenotypic variation, respectively. The remaining epistatic interaction (E-PUS3^PP^-E-PUS6^PP^) had both genetic and E-QE interaction effects, and the percentage of phenotypic variation obtained was 1.1% and 0.9% for genetic and E-QE interaction effects, respectively. A detailed description of the digenic epistatic interaction analysis is shown in Table 7.

**Table 7:** Epistatic QTLs (E-QTLs) and E-QTL x Environment (E-QE) effects detected for popping traits using multi-environment analysis

**E-QTLi**^**a**^	**Marker interval**	**LG (position)**^**b**^	**E-QTLj**^**a**^	**Marker interval**	**LG (position)**	**AA**^**c**^	***h***^**2**^**(aa)**^**d**^	**E-QE AAE**^**e**^	***h***^**2**^**(aae)**^**f**^
Popping dimension index (PDI)									
E-PDI3^PP^	IAC24 -BM187	3 (71.0-84.9)	E-PDI7^PP^	BM185 -BMc294	7 (24.2-36.6)	−2.98^***^	2.7	ns	
Expansion coefficient (EC)									
E-EC3^PP^	BMc259 -IAC24	3 (67.8-71.0)	E-EC9^PP^	PV-at007 -BMc184	9 (60.9-70.2)	1.62^*^	0.2	ns	
E-EC5^PP^	E32M60-100 -BM175	5 (40.6-46.1)	E-EC7^PP^	BM185 -BMc294	7 (71.0-84.9)	−7.59^***^	2.6	ns	
Percentage of unpopped seeds (PUS)									
E-PUS3^PP^	BMc259 -IAC24	3 (67.8-71.0)	E-PUS6^PP^	E40M60-91 -E45M50-50	6 (5.0-5.8)	−3.87^***^	1.1	−4.61^*^ AAE4	0.9
E-PUS3^PP^	BMc259 -IAC24	3 (67.8-71.0)	E-PUS7^PP^	BMc294 -BMc248	7 (36.6-38.1)	5.47^***^	0.6	ns	
E-PUS5^PP^	BM138 -E32M60-100	5 (39.8-40.6)	E-PUS6^PP^	E40M60-91 -E45M50-50	6 (5.0-5.8)	−3.62^***^	1.8	ns	

^a^ E-QTLi and E-QTLj are the two QTLs involved in epistatic interaction.

^b^ Linkage group and the estimated confidence interval of QTL position in brackets (in Kosambi cM).

^c^ Estimated additive by additive epistatic effect. Positive values indicate that alleles from PHA1037 have a positive effect on the traits, and negative values indicate that positive effect on the traits is due to the presence of the alleles from PMB0225.

^d^ Percentage of the phenotypic variation explained by additive by additive epistatic effects.

^e^ Predicted additive by additive epistatic effect by environment interaction effect. AAE1, AAE2, AAE3, AAE4, and AAE5: epistasis associated with environments LD09, SD09, LD10, SD10, and LD11, respectively. The meaning of sign values is described in the third footnote (^c^).

^f^ Percentage of the phenotypic variation explained by additive by additive epistatic effect by environment interaction effect.

^*^P ≤ 0.05, ^**^P ≤ 0.01, ^***^P ≤ 0.001. Only significant effects are listed. ns = No significant effects on the five environmental conditions evaluated.

## Discussion

The main goal of the current study was to unravel the genetic architecture of popping ability in nuña bean. Thus, popping traits related to changes in the physical structure of seeds have been analysed on the basis of their similarity to popcorn, whose cotyledons also expand when dry grains are heated. Genetic analysis performed indicated that popping ability traits show a polygenic inheritance, making this the first work to report the genetic control of these traits in common bean. Transgressive segregation was observed for popping traits, suggesting that combinations of alleles from both parents have effects in the same direction; in fact, not only PHA1037 but also PMB0225 bear alleles with a positive effect on popping ability, a finding backed up by QTL analyses. Since transgressive segregation relies on additive genetic variation, the extreme phenotypes can be maintained and fixed through artificial selection, providing the potential for improvement of popping ability. Furthermore, the analysis of variance showed that although the genotype x environment interaction affects popping ability, this effect is fairly uniform across all genotypes, and it does not seriously compromise genotypic main effects, making progress from selection feasible.

A comprehensive QTL analysis was performed to detect single-locus QTLs, epistatic QTLs and their environment interactions on a newly created genetic linkage map. This map was constructed for an Andean intra-gene pool cross involving PMB0225 (dry bean) and PHA1037 (popbean) parents. Despite the morphological diversity observed in the Andean intra-gene pool, the low genetic polymorphism existing in this common bean germplasm hinders the development of genetic linkage maps [31,33,51,52]. Our results confirmed a low polymorphism between the Andean parents, thus the overall polymorphism rate detected was 8.3%. Likewise, Blair et al. [33] screened a total of 700 SSR markers on the Andean parents G21242 and G21078, but only 74 mappable markers were found in that survey resulting in a polymorphism rate of 10.6%, comparable to the level of polymorphism here reported (i.e. 10.2% for SSR markers). However, Cichy et al. [31] found a moderate SSR polymorphism of 30% between the Andean parents used to generate a G19833 x AND696 RIL mapping population.

Interestingly, the genetic map described in this work shares 55 SSR markers with previously published common bean maps [26,31-33,45-50]. In fact, linkage associations have been found in terms of SSR marker mapping in the present map and previous maps, while the collinear order of the commonly mapped SSR loci has been generally observed although some inversions affecting SSR markers located close to one another have been detected. Since these loci were distributed throughout all LGs, they would permit the alignment of homologous LGs between maps and facilitate marker transfer across populations as well as between related species. Hence, these shared markers could be used as anchor points for map merging and syntenic analysis such as Galeano et al. [35] have recently reported for the consensus Mesoamerican intra-gene pool map.

The genetic linkage map developed herein includes 193 loci (85 AFLP, 95 SSR, and 13 SNP markers) across 12 LGs that cover a genetic distance of 822.1 cM, with an average of 4.3 cM per marker. Prior to this work, two Andean maps have been described for QTL analysis; the map depicted by Cichy et al. [31] included 167 loci that spanned a total map length of 1105 cM with an average marker density of 6.6 cM per locus. On the other hand, the map constructed by Blair et al. [33] contained 118 loci with a total map length of 726.0 cM and a mean marker density of 6.2 cM per locus. Therefore, compared to previous Andean maps [31,33], the genetic map here developed shows a suitable marker density and genome coverage, which has permitted the first identification of popping ability QTLs.

Three closely related popping traits such as PDI, EC, and PUS have been analysed and the reliability of the QTLs associated to these traits has been enhanced by using several software programs, which decreased the risk of detecting false positive and negative QTLs [53-55]. Therefore, single and multi-environment QTL analyses were performed to dissect the genetic architecture of popping ability in nuña bean. In summary, a total of nineteen single-locus QTLs were identified by MapQTL and QTLNetwork. Eleven of the fourteen QTLs identified by QTLNetwork were also detected by MapQTL; thus, the two independent approaches converged on the identification of common single-locus QTLs for PDI (PDI3^PP^, PDI5^PP^, PDI6^PP^, and PDI7^PP^), EC (EC3^PP^, EC5^PP^, and EC7^PP^), and PUS (PUS3^PP^, PUS5^PP^, PUS6^PP^, and PUS7^PP^). The percentage of phenotypic variation explained by the single-locus QTLs identified by MapQTL for popping traits was comparatively higher than that of the QTLNetwork. The results of multi-environment analyses showed that genetic main effects were sometimes subject to environmental modification; this could explain why we obtained a lower phenotypic variance using QTLNetwork software. Even so, for multi-environment analyses, the percentage of phenotypic variance attributable to genetic effects was as expected for a complex trait, which is governed by several small effect QTLs/genes located in different genomic regions, and where the environment interactions play an important role. Therefore, the four common single-locus QTLs detected for PDI and PUS together explained 22.2 and 21.8% of the phenotypic variance, respectively. Regarding EC, the three common QTLs explained 11.7% of the phenotypic variance in the RIL population. In addition, it was interesting to find that the common QTLs (detected with both software programs) not only were consistent over environments, but they also co-localized with QTLs for the analysed popping traits.

Overall, significant positive correlations between PDI and EC and negative correlations among PUS, and PDI and EC, together with the detection of co-localized QTLs for PDI, EC, and PUS on LGs 3, 5, 6, and 7, suggested that QTLs for popping ability are not evenly dispersed throughout the genome but rather are clustered in several genomic regions. The QTLs sign values of additive effects corresponded to the significant genotypic correlations observed among the three popping traits. Thus, the co-localized QTLs located on LGs 3, 5 and 6 showed positive (alleles from PHA1037) and negative (alleles from PMB0225) values of additive effects for PDI and EC, and PUS, respectively. Meanwhile, the opposite sign values of additive effects were found for the co-localized QTLs located on LG 7, which indicated that PMB0225 also contributes positively to popping ability. To date, research into popping ability has focused on popcorn. Thus, Babu et al. [21] detected four QTLs for popping expansion volume, five for flake volume, and five for percentage of unpopped kernels, and revealed QTLs in overlapping or mostly adjoining regions in the same chromosomes affecting two or three popping traits. Likewise, Li et al. [8] evaluated three important traits for popcorn (i.e. popping volume, flake size, and popping rate), and six chromosome regions were found to control two or three popping traits simultaneously. Hence, as in nuña bean, the detection of co-localized QTLs for popping traits suggested that either pleiotropic QTLs controlled several popping traits, or tightly linked QTLs for different traits are present together in the same genomic regions. The issue of pleiotropy versus tight linkage of QTLs may be resolved in the future through fine mapping of the target genomic regions.

Epistatic effects are often involved in complex traits, but they are difficult to confirm because of their usually small effects and environmental interactions. Genetic models for QTL mapping assuming no epistasis could lead to biased estimation of QTL parameters, and subsequently result in considerable loss of response in MAS. In popcorn, Li et al. [8] carried out a preliminary epistatic analysis and detected thirteen pairs of digenic interactions for popping ability. In the present work, several epistatic interactions were found involving all of the evaluated popping traits. A total of ten E-QTLs, involved in six epistatic interactions, were detected, and only one epistatic interaction for PUS showed significant E-QE interaction effect. Although the phenotypic variation explained by each epistatic interaction was found to be small, it is interesting to note that the genomic regions located on LGs 3, 5, 6 and 7 not only harbor QTLs that have individual genetic effects, but are also involved in epistatic interactions. Therefore, QTL analysis revealed that popping ability of nuña bean is controlled by several QTLs, which have only individual additive effects, or may also be involved in epistatic or environmental interactions, indicating that popping is inherited as a polygenic trait, and that epistasis could play a key role.

Nowadays, popping of common bean is considered an interesting agronomic trait, since it allows greater diversification of this crop as well as the commercialization of nuña bean as a new snack product. In popcorn, selection for increased expansion coefficient has been successfully achieved given its high heritability value [20,21]. Similarly, Vorwald and Nienhuis [56] estimated that the narrow sense heritability values of fully expanded seeds after popping (popping percentage) and expansion coefficient in nuña bean were relatively high, 0.87 ± 0.07 and 0.74 ± 0.09, respectively. The broad sense heritability values calculated in the present work were moderate, suggesting that genetic gain could be obtained for popping ability in this legume species. The introgression of popping and the development of new day-length insensitive popbean cultivars would require genetic tools which facilitate efficient genotyping selection. Conventional phenotype selection methods for popping traits are laborious and time-consuming; consequently, MAS provides an efficient and cost-effective alternative that accelerates the selection of interesting genotypes. However, MAS approaches have been difficult to apply in the case of complex traits such as popping ability, because individual QTLs have small genetic effects which in many cases are also environmentally modulated. Consequently, the identification of potential candidate QTLs for MAS is crucial. Based on the results obtained in our study, the co-localized QTLs located on LGs 3, 5, 6, and 7 are good candidates for MAS, since they showed stability across significantly correlated traits, while also sharing QTLs for more than one trait, and they could be manipulated simultaneously in breeding programs. Breeding of nuña cultivars would require adapting them to temperate regions, and for this purpose it is important to improve their insensitivity to photoperiod. The *Ppd* gene regulates photoperiod sensitivity and it is located on LG 1 [57], while popping QTLs are located on LGs 3, 5, 6, and 7. Therefore, the use of QTL marker assisted selection would facilitate the introgression of photoperiod insensitivity without loss of popping ability. QTL pyramiding approach would also permit the combination of QTL alleles with positive effects for popping ability on a day-length insensitive genotype through molecular breeding, thus overcoming the main drawback for the production and commercialization of nuña beans in temperate regions. In this research, some RILs showed popping expansion ability and flowered independently of photoperiod conditions. These lines constitute an interesting breeding goal, and they will hopefully allow researchers to isolate the genes and to understand the molecular and physiological mechanisms underlying agronomic traits which are relevant for the genetic improvement of nuña beans.

## Conclusions

We have developed a novel Andean genetic linkage map, which has permitted the first identification of popping ability QTLs in common bean. Our results revealed that popping ability of nuña bean is controlled by several QTLs, which have only individual additive effects or may also be involved in epistatic or environmental interactions, indicating that popping is inherited as a polygenic trait, and that epistasis could play a key role in its genetic control. Individual and multi-environment QTL analyses detected a total of nineteen single-locus QTLs, most notably those co-localized for the three popping ability traits placed on LGs 3, 5, 6, and 7. These QTLs showed an individual effect and also participated in epistatic interactions. Consequently, the co-localized QTLs for popping expansion response are useful tools for MAS breeding programs intended to improve production and adaptation of nuña bean cultivars. The results here reported can contribute towards the diversification of the nuña bean crop, which is becoming increasingly relevant as a new food product in the agro-food industry due to its nutritional and healthy properties.

## Methods

### Population development

**Figure 2:**
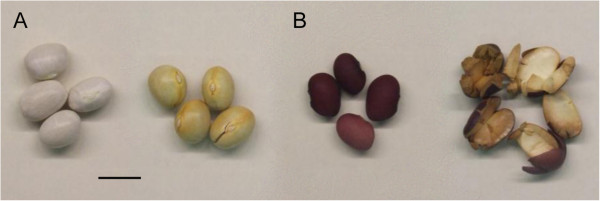
**Unpopped and popped seeds of the parental genotypes.** Both PMB0225 (**A**) and PHA1037 (**B**) belong to the Andean gene pool of common bean. *Scale bar* 1 cm.

Parents included two genotypes from the Andean gene pool, PMB0225 and PHA1037, belonging to Nueva Granada and Peru races, respectively. PMB0225 is a Spanish improved cultivar resistant to the bean common mosaic virus, which shows indeterminate erect growth habit type II, white flowers and large seeds. PHA1037 is a photoperiod-sensitive nuña popbean germplasm accession from Bolivia that has purple flowers and large red seeds, and possesses an indeterminate climbing growth habit type IV (Figure 2). Both parents were chosen to facilitate the introgression of the popping trait in common bean cultivar adapted to temperate areas. Individual plants of a F_2_ segregating population generated from the cross of PMB0225 × PHA1037 were selfed to develop 185 F_7_ recombinant inbred lines (RILs) by single-seed descent.

### Experimental design

The 185 RILs and both parents were evaluated for popping seed traits using 15 plants per genotype in five greenhouse environments over three consecutive years (2009, 2010 and 2011). Plants were grown under long-day (LD) and short-day (SD) natural photoperiod conditions with average day and night temperatures of 25°C and 20°C, respectively. Sowing dates of LD experiments were February 20, 2009 (LD09 code), March 15, 2010 (LD10 code), and April 27, 2011 (LD11 code), while sowing dates of SD experiments were August 15, 2009 (SD09 code) and September 21, 2010 (SD10 code). For all environments the experiments were conducted in a randomized complete block design with two or three replicates of single row plots (3.0 × 0.8 m). Each plot was sown with two seeds per hill and adjusted to a crop density of about 30,000 plants/ha. Pods were harvested when they were completely dried. Seeds were removed and cleaned using a mechanical thrasher followed by hand cleaning and winnowing. Then, seeds were stored at 5°C for about one month before initiation of the present research.

### Trait measurements

Three popping component traits have been measured: popping dimension index (PDI), popping expansion coefficient (EC), and percentage of unpopped seeds (PUS). Samples of 50 seeds from each treatment, replicate and environment, were popped in a Palson Denver popcorn maker (1200 W, 230 V, 50 Hz) for 150 s. Seed was considered fully popped when cotyledons had expanded sufficiently to shed the seed coat, and unpopped or partially popped when the seed coat failed to crack or no expansion of the cotyledons was observed. Seed dimensions were scored for each individual seed: length (mm) was measured as the longest distance across the seed parallel to the hilum, height (mm) as the longest distance perpendicular to length, and width (mm) as the longest distance across the hilum seed. Seed length, width and thickness before and after popping were determined from a random 10-seed sample, and PDI was recorded as [(∑popped seed dimensions-∑unpopped seed dimensions)/∑unpopped seed dimensions) x 100]. Each 50-seed sample was placed in a graduated cylinder and distilled water was added to a total volume of water and seeds of 100 mL. The total volume of water added was subtracted from the total volume to give the unpopped seed volume (unPV). The seeds were drained and patted dry with paper toweling and immediately popped to minimize absorption of water by the seeds. The volume of seeds after popping (PV) was obtained using a procedure similar to that used for unpopped seeds. The EC was defined as [(PV-unPV)/unPV] x 100. The PUS was calculated as the proportion of 50 seeds that were unpopped.

### Statistical data analysis

Analysis of variance with the Generalized Linear Model (GLM) procedure was applied to analyse phenotypic data using the SAS Software [58]. Single degree-of-freedom orthogonal contrasts between parents were calculated to show significant differences. For each trait and environment, mean value, standard deviation and range of variation were calculated. The phenotypic correlation coefficients between PUS and EC, and PDI, were estimated by using PROC CORR [58]. Broad sense heritability (H^2^) was estimated as H^2^ = σ^2^G/(σ^2^e/re + σ^2^GE/e + σ^2^G), where σ^2^G is the estimate of genotypic or RIL variance, σ^2^e is the estimate of error variance, σ^2^GE is the estimate of genotype x environment interaction variance, r is the number of replicates per environment, and e is the number of environments. The genetic components of variance were estimated with the MIXED procedure of SAS software [58].

### DNA extraction and molecular marker analyses

Total genomic DNA was isolated from young leaves as described by Doyle and Doyle [59]. DNA was kept in sterile water, visualized after electrophoresis in 1% agarose gels in 1X SB buffer (10 mM sodium boric acid), and quantified by comparison with DNA standards (Lambda phage DNA digest with *Hin*dIII; Invitrogen Life Technologies). DNA was diluted in sterile water to a stock concentration of 5–10 ng/μL and stored at −20°C for use in PCR analysis.

Analysis of AFLP markers was carried out according to the procedure described by Vos et al. [60], with some modifications [61]. A total of 500 ng of genomic DNA was digested with 5 U of *Mse*I and *Eco*RI enzymes for 2 h at 37°C in a final volume of 40 μL. The DNA fragments were ligated to appropriate adapters via addition of 1 U of T4-DNA Ligase (Roche) and incubated for 1 h at 37°C. The pre-amplification reactions were performed in a volume of 20 μL using A as the selective nucleotide for the Eco primer (Eco + A) and four different Mse primers (Mse + A, Mse + C, Mse + G, and Mse + T). The PCR cycling parameters were 20 cycles at 94°C for 30 s, 56°C for 60 s, and 72°C for 60 s. Subsequent PCR amplifications were performed with primers that included three selective bases in their sequences. To detect AFLP fragments, Eco primers were labelled using a fluorescent dye (FAM, NED, PET or VIC), and the following PCR cycling parameters were used for selective amplifications: an initial cycle at 94°C for 30 s, 65°C for 30 s, and 72°C for 60 s. During the next 12 cycles, the annealing temperature was lowered by 0.7°C per cycle. The temperature conditions for the next 23 cycles were 94°C for 30 s, 56°C for 30 s, and 72°C for 60 s. The PCR products of selective amplifications were separated by capillary electrophoresis using a DNA sequencer (ABI PRISM® 3130 XL Genetic Analyzer, Applied Biosystems, USA). An internal size marker, GeneScan 500 LIZ (35–500 bp; Applied Biosystems) was added, allowing the co-loading of different labelled reactions. Data regarding selectively amplified DNA fragments were analysed with GeneMapper Software 3.7 (Applied Biosystems). Each AFLP marker name included a Keygene primer code (http://www.wheat.pw.usda.gov/ggpages/keygeneAFLPs.html) followed by the fragment size in base pairs.

Different sets of SSR and SNP markers previously reported (references are shown below) were tested for polymorphism in the parental genotypes, and polymorphic loci were used for the construction of the genetic linkage map. SSR markers were named according to the respective authors (IAC-, [49,62,63]; ATA-, [45]; BM-, GATS-, [64,65]; BMb-, [47]; BMc-, [46,52]; BMd-, [66]; PV-, [67]; PVBR-, [68,69]; PVEST-, [50]; PvM-, FJ-, [26,70]). PCR reactions were carried out following the protocols described in the publications mentioned above, although PCR conditions were changed for some SSR markers. Data analysis of the SSR markers was performed by using either gel electrophoresis or capillary electrophoresis in an ABI PRISM® 3130 XL Genetic Analyzer (Applied Biosystems, USA). SNP markers tested in our mapping population were designated as BSNP- [71], Leg- [72], and SNP- [73]. High resolution melting technology (HRM) was employed to analyse the SNP markers using a LightScanner instrument (Idaho Technology), following the protocols described by Montgomery et al. [74].

### Linkage map construction and QTL analyses

JoinMap® 4.0 software [75] was used to generate the linkage maps. Marker data were assigned to LG using a minimum logarithm of odds ratio (LOD) score of 6.0, and a recombination frequency value of 0.3. The Kosambi map function [76] was used to calculate the genetic distance between markers. The LGs were designated according to Pedrosa-Harand et al. [44].

Candidate QTL regions for popping traits were identified by using two different mapping software packages, MapQTL® 5.0 [77] and QTLNetwork 2.0 [78]. Interval mapping and multiple QTL model (MQM) mapping were used to detect single-locus QTLs for each environment separately by MapQTL. Thus, once potential QTLs were detected by interval mapping analysis, markers with higher LOD scores were selected as cofactors and tested using the automatic cofactor selection procedure (default *P* value cut off for elimination of a cofactor set of 0.02). Using the set of selected cofactors, MQM mapping analyses were carried out. A permutation test (1000 cycles) was used to determine the LOD threshold score at which the QTL was deemed to be present in a particular genomic region with a confidence interval of 95%. In addition, QTLNetwork software was used to identify single locus QTLs, epistatic QTLs (E-QTL) and their environment interaction effects (QTLs x Environment, QE; and E-QTLs x Environment, E-QE) across environments. The mixed-model based composite interval mapping method (MCIM) was carried out for one-dimensional genome scan to detect putative QTLs and their environment interactions, and for two-dimensional genome scan to identify epistatic effects. An experimental-wise significant level of 0.05 was designated for candidate interval selection, putative QTL detection, and QTL effect. Both testing and filtration window size were set at 10 cM, with a walk speed of 1 cM. The critical *F*-value to declare putative QTLs was determined by 1000 permutation test. The effects of QTLs and environment interactions were estimated by Markov Chain Monte Carlo method [79]. QTLs with only genetic effects indicated that these were expressed in the same way across environments. In addition, QTLs with environment interaction effects suggested that their expressions were environmentally dependent. The detected QTLs were designated as recommended by Miklas and Porch [80]. The genetic map and the QTLs detected were drawn using the MapChart 2.2 software [81].

## Abbreviations

AFLP: Amplified fragment length polymorphic; EC: Expansion coefficient; E-QE: E-QTL x Environment effect; E-QTL: Epistatic QTL; LD: Long-day; LGs: Linkage groups; LOD: Logarithm of odds ratio; MAS: Marker assisted selection; MCIM: Mixed-model based composite interval mapping; MQM: Multiple QTL model mapping; PDI: Popping dimension index; PUS: Percentage of unpopped seeds; QE: QTL x Environment effect; QTL: Quantitative trait loci; RFLP: Restriction fragment length polymorphic; RILs: Recombinant inbred lines; SD: Short-day; SNP: Single nucleotide polymorphism; SSR: Simple sequence repeats.

## Authors’ contributions

FY-L carried out genetic and molecular marker analyses, developed the genetic map and drafted the manuscript. MS developed the recombinant inbred line population, performed the field trials and carried out the phenotypic evaluation of plant material, and collaborating in writing the manuscript. CC and MG-A collaborated in marker genetic analyses and contributed to data analysis. MDLF collaborated in the phenotypic analysis of segregating population and the selection of microsatellite markers. JC participated in the design of genotyping experiments, supported mapping methodologies and contributed to a critical review of the manuscript. AMDR provided significant information about the plant material, collaborated in the analysis of the phenotypic and genetic data and reviewed the manuscript. RL conceived the project, planned the research work, assisted in analysis and interpretation of the data, and edited the manuscript. All authors have read and approved the final manuscript.
